# Experimental partitioning of halogens and other trace elements between olivine, pyroxenes, amphibole and aqueous fluid at 2 GPa and 900–1,300 °C

**DOI:** 10.1007/s00410-013-0902-5

**Published:** 2013-06-19

**Authors:** Alessandro Fabbrizio, Roland Stalder, Kathrin Hametner, Detlef Günther, Katharina Marquardt

**Affiliations:** 1 Institute of Mineralogy and Petrography, University of Innsbruck, Innrain 52f, 6020 Innsbruck, Austria; 2 Lab of Inorganic Chemistry, ETH Zürich, Wolfgang-Pauli-Str. 10, 8093 Zurich, Switzerland; 3 Deutsches GeoForschungsZentrum, Section 3.3, Telegrafenberg, 14473 Potsdam, Germany

**Keywords:** Halogens, Trace element partitioning, Mantle, Defects, TEM

## Abstract

We present new partition coefficients for various trace elements including Cl between olivine, pyroxenes, amphibole and coexisting chlorine-bearing aqueous fluid in a series of high-pressure experiments at 2 GPa between 900 and 1,300 °C in natural and synthetic systems. Diamond aggregates were added to the experimental capsule set-up in order to separate the fluid from the solid residue and enable in situ analysis of the quenched solute by LA–ICP–MS. The chlorine and fluorine contents in mantle minerals were measured by electron microprobe, and the nature of OH defects was investigated by infrared spectroscopy. Furthermore, a fluorine-rich olivine from one selected sample was investigated by TEM. Results reveal average Cl concentrations in olivine and pyroxenes around 20 ppm and up to 900 ppm F in olivine, making olivine an important repository of halogens in the mantle. Chlorine is always incompatible with Cl partition coefficients *D*_Cl_^olivine/fluid^ varying between 10^−5^ and 10^−3^, whereas *D*_Cl_^orthopyroxene/fluid^ and *D*_Cl_^clinopyroxene/fluid^ are ~10^−4^ and *D*_Cl_^amphibole/fluid^ is ~5 × 10^−3^. Furthermore, partitioning results for incompatible trace element show that compatibilities of trace elements are generally ordered as *D* ^amph/fluid^ ≈ *D* ^cpx/fluid^ > *D* ^opx/fluid^ > *D* ^ol/fluid^ but that *D* ^mineral/fluid^ for Li and P is very similar for all observed silicate phases. Infrared spectra of olivine synthesized in a F-free Ti-bearing system show absorption bands at 3,525 and ~3,570 cm^−1^. In F ± TiO_2_-bearing systems, additional absorption bands appear at ~3,535, ~3,595, 3,640 and 3,670 cm^−1^. Absorption bands at ~3,530 and ~3,570 cm^−1^, previously assigned to humite-like point defects, profit from low synthesis temperatures and the presence of F. The presence of planar defects could not be proved by TEM investigations, but dislocations in the olivine lattice were observed and are suggested to be an important site for halogen incorporation in olivine.

**Electronic supplementary material:**

The online version of this article (doi:10.1007/s00410-013-0902-5) contains supplementary material, which is available to authorized users.

## Introduction

Mantle minerals such as olivine, ortho- and clinopyroxene can host up to several hundreds ppm water (Rauch and Keppler 2002; Hirschmann et al. 2005; Stalder et al. 2005, 2008; Grant et al. 2007a; Tenner et al. 2009). The incorporation mechanism of OH in mantle minerals is important for understanding the rheology, partial melting, diffusion, electrical conductivity, seismic wave speeds and attenuation of the mantle and has been studied intensely in the previous decades (e.g., Bell and Rossman 1992; Hirth and Kohlstedt 1996; Karato and Jung 1998). In contrast, the role of the halogens F and Cl in the mantle is not well investigated. From geochemical considerations (Cl/K and F/P ratios of melt inclusions from mid-ocean ridge basalts and the estimated K and P contents of the upper mantle), the abundance of chlorine in the mantle is estimated to approximately 1 ppm and that of fluorine to 16 ppm (Saal et al. 2002). Despite their low abundances in the mantle, halogens exert a significant role on the genesis and evolution of magmas. Halogens play an important role in volcanic and related magmatic systems (Aiuppa et al. 2009; Baker and Balcone-Boissard 2009; Webster et al. 2009). Halogens are separated from magma in the Earth’s crust during cooling and crystallization (Carroll and Webster 1994) and exert significant impact on the genesis of hydrothermal systems (e.g., Reed 1997) and on the transport of ore-forming metals (Yardley 2005; Vigneresse 2009).

Halogen solubilities in nominally anhydrous mantle minerals at mantle conditions are scarcely documented, but seem to be much higher than observed in natural samples (Beyer et al. 2012). Experimental results in the system Mg_2_SiO_4_–MgF_2_ (Bromiley and Kohn 2007) reveal up to 0.45 wt% F in forsterite, but no apparent correlation between F and Mg concentrations in the olivine could be established, and the incorporation mechanisms could not be solved. In contrast, Cl solubilities are much lower. Chlorine concentrations for forsterite and enstatite in fluid-bearing systems cluster around 3 ppm in the system forsterite–enstatite–pyrope–H_2_O–MgCl_2_ (Bernini et al. 2013). In contrast, Cl concentrations around 20 ppm were observed in the system MgO–SiO_2_–H_2_O ± TiO_2_ ± Al_2_O_3_ under similar *P*–*T* conditions (Fabbrizio et al. 2013), where the enhanced Cl solubilities in forsterite in the presence of TiO_2_ suggest a Cl incorporation in forsterite via the stabilization of defects that are stabilized by Ti, e.g., humite-type defects. Ti plays an important role in the formation of hydroxyl point defects in olivine associated with trace Ti substitutions (Berry et al. 2005), and planar humite type associated with Ti has been detected in olivine from different localities (Kitamura et al. 1987; Drury 1991; Risold et al. 2001; Hermann et al. 2007) by transmission electron microscopy (TEM). Both kinds of defects are able to host halogens that substitute some of the OH. In addition to planar and point defects, the growth of nanometer-sized Ti-clinohumite nuclei during hydrous alteration in olivine at 8 GPa and 1,300 K has been described (Wirth et al. 2001), otherwise no detailed TEM studies have been carried out to confirm the presence of humite-type defects in synthetic olivine. Olivine crystals that have been investigated by TEM and revealed humite-type planar defects exhibit IR absorption bands at 3,564 and 3,394 cm^−1^, identical in energy to absorption bands in Ti-clinohumite (Hermann et al. 2007). In contrast, Ti-related OH point defects in olivine cause IR absorption bands at 3,525 and 3,572 cm^−1^ (Berry et al. 2005). Partitioning experiments at upper mantle conditions between nominally halogen-free minerals and coexisting basaltic melts both in natural and in synthetic systems show that (1) F and Cl are always incompatible in mantle minerals, (2) the compatibilities are generally ordered as *D* ^Cpx/melt^ > *D* ^Opx/melt^ > *D* ^Grt/melt^ > *D* ^Ol/melt^ > *D* ^Plag/melt^ and (3) F is less incompatible than Cl (Beyer et al. 2012; Dalou et al. 2012).

In this study, we present new experimental results derived from synthesis experiments at 2 GPa and 900–1,300 °C for F and Cl incorporation in olivine, ortho- , clinopyroxene, and amphibole and mineral/fluid partition coefficient for Cl in the system MgO–SiO_2_–H_2_O ± TiO_2_ ± NaCl ± MgF_2_ ± CaF_2_ and in natural composition doped with TiO_2_, NaCl or CaF_2_. A combination of electron microprobe and LA–ICP–MS was used to analyze Cl in the resulting phases. The quality and quantity of OH defects in olivine were characterized by IR spectroscopy, and transmission electron microscopy (TEM) was used to investigate the relations between the OH-related IR absorption bands and humite-type defects.

## Experimental and analytical methods

### Starting materials and sample preparation

The starting materials for the experimental runs consisted of a natural peridotite from Stöppling/Germany (details concerning petrography see Oehm et al. 1983; composition was determined by XRF: SiO_2_ = 44.2 wt%; Al_2_O_3_ = 1.3 wt%; Fe_2_O_3_ = 9.4 wt%; MnO = 0.1 wt%; MgO = 43.6 wt%; CaO = 1.3 wt%) and of a synthetic MSH (MgO = 40.3 wt%; SiO_2_ = 41.7 wt%; H_2_O = 18 wt%) powder both doped with TiO_2_, NaCl, CaF_2_ or MgF_2_ (Table 1).

**Table 1:** Starting mixtures, experimental conditions^a^ and phase relations of the run products

Run	Starting material	*T* (°C)	Time (h)	Run products identified
Cl-22	Peridotite + 0.3 % TiO_2_ + 22 % NaCl	1,300	24	ol, salt, fluid
Cl-23	Peridotite + 0.3 % TiO_2_ + 22 % NaCl	1,200	48	ol, salt, fluid
Cl-24	Peridotite + 0.3 % TiO_2_ + 22 % NaCl	1,100	72	ol, opx, salt, fluid
Cl-25	Peridotite + 0.3 % TiO_2_ + 22 % NaCl	1,000	96	ol, opx, cpx, salt, fluid
Cl-26	Peridotite + 0.3 % TiO_2_ + 22 % NaCl	900	144	ol, opx, cpx, amph, salt, fluid
Cl-22^b^	Peridotite + 0.3 % TiO_2_ + 22 % NaCl	1,300	24	ol, salt, fluid
Cl-23^b^	Peridotite + 0.3 % TiO_2_ + 22 % NaCl	1,200	48	ol, salt, fluid
Cl-24^b^	Peridotite + 0.3 % TiO_2_ + 22 % NaCl	1,100	72	ol, opx, salt, fluid
Cl-25^b^	Peridotite + 0.3 % TiO_2_ + 22 % NaCl	1,000	96	ol, opx, cpx, salt, fluid
Cl-26^b^	Peridotite + 0.3 % TiO_2_ + 22 % NaCl	900	144	ol, opx, cpx, amph, salt, fluid
F-2	MSH + 0.3 % TiO_2_ + 1 % CaF_2_	1,300	24	fo, en, salt, fluid
F-3	MSH + 0.3 % TiO_2_ + 1 % CaF_2_	1,200	48	fo, chu, en, salt, fluid
Cl–F-1	MSH + 22 % NaCl + 2 % MgF_2_	1,200	48	fo, en, salt, fluid
F-1	MSH + 0.3 % TiO_2_ + 1 % CaF_2_	1,000	96	fo, en, di, tr, tlc^d^, salt, fluid
F-5^c^	Peridotite + 0.3 % TiO_2_ + 22 % NaCl + 2 % CaF_2_	1,300	24	ol, sp, salt, fluid
F-4^c^	Peridotite + 0.3 % TiO_2_ + 22 % NaCl + 2 % CaF_2_	1,300	24	ol, salt, fluid
F-6	Peridotite + 0.3 % TiO_2_ + 22 % NaCl + 2 % CaF_2_	1,300	24	ol, opx, salt, fluid
F-7	Peridotite + 0.3 % TiO_2_ + 22 % NaCl + 2 % CaF_2_	1,100	72	ol, opx, salt, fluid

*Ol* olivine, *opx* orthopyroxene, *cpx* clinopyroxene, *amph* amphibole, *fo* forsterite, *en* enstatite, *chu* clinohumite, *di* diopside, *tlc* talc, *tr* tremolite, *sp* spinel

^a^All runs were performed at a pressure of 2 GPa

^b^Experiment performed with diamond trap

^c^Capsule lined with Ni (F-4) and Fe (F-5) foil

^d^Supposed by mixed analysis fo + tlc (Table 3)

Starting mixtures were prepared by grounding the peridotite and the synthetic Mg(OH)_2_–SiO_2_ mixture in an agate mortar under ethanol for 1 h and subsequently in an automatic milling machine for 1 h. The doping agents were then added to the starting mixtures in the desired amount. In addition, barium was added as BaTiO_3_ to the peridotite mixture. Barium is highly incompatible in mantle minerals. Therefore, it resides preferably in the fluid or melt phase and can be used as internal standard for quantification of LA–ICP–MS analyses. The doped starting mixtures were ground again in the agate mortar under ethanol for 1 h to achieve chemical homogeneity.

As sample containers, we used Pt capsules with an outer (inner) diameter of 3.0 (2.6) mm. For experiments with the diamond trap, a Pt ring with an inner diameter of 2.1 mm and a height of 1 mm was inserted into the Pt capsule. Then, ~6 μl H_2_O was added to the capsule, corresponding to 15 wt% of the starting powder + water mixture. The capsule was then filled with 6–7 mg of synthetic diamond crystals (grain size 20 μm) kindly provided by Servsix GmbH (Karlstein, Germany) and with 35–55 mg of the starting material powder. For experiments without the diamond trap, the set-up of the capsule was the same except for the Pt ring and for the diamond crystals that were not used. For experiments with the MSH mixtures, 2 μl H_2_O was added to the capsule. The capsule was welded while cooled in water, and the weight of the capsule was compared before and after welding to ensure that no water was lost during welding. The sealed capsule was squeezed to a final cylindrical shape with a length of 5.5–6.5 mm and then held at 120 °C for 24 h, in order to check whether the capsule was sealed properly.

### Experiments

Experiments were carried out in an end-loaded piston-cylinder apparatus at 2 GPa and temperatures in the range of 900–1,300 °C for durations between 1 and 6 days at Innsbruck University (Table 1). The concentric cylindrical high-pressure assemblies consist (from outside to inside) of a lead foil wrapped around a talc cell, a Pyrex cylinder, a graphite furnace and inner pieces of crushable alumina sleeves. In two experiments, the interior walls of the capsules were lined with Ni (run F-4) and Fe foil (run F-5) to control the *f*O_2_ (Table 1). At pressure and temperature of interest (2.0 GPa and 1,300 °C), the calculated log*f*O_2_ for the controlling reaction Fe–FeO is ~3 log units below the QFM buffer (QFM-3) and for the reaction Ni–NiO is ~1.5 log units above the QFM buffer (QFM + 1.5) (O’Neill 1988; O’Neill and Pownceby 1993). Run temperatures were controlled within ~1 °C of the set point using Pt/Pt_90_Rh_10_ thermocouples accurate to within ±3 °C. The range in temperature across the length of the capsules during the experiments is estimated to be less than 20 °C. The experimental charge is placed in the hotspot of the experimental assembly to prevent unwanted crystal growth within the diamond trap (Aerts et al. 2010). No pressure correction was applied to the EMF. The power output was monitored during the entire duration of all runs in order to ensure that no temperature drift occurred. Each experiment was initiated by gently increasing the sample pressure to approximately 0.25 GPa followed by a heating to 1,400 °C at a rate of 15 °C/min and a simultaneous pressure increase until the final pressure was reached. To grow relatively large, high-quality crystals necessary for IR spectroscopy, synthesis experiments were heated to 1,400 °C and then slowly cooled to final temperature at a rate of 6 °C/h. Depending on the temperature, the experimental run time was in the range 24–144 h (Table 1), corresponding to the time needed to achieve equilibrium in similar systems under similar conditions (Stalder et al. 2001; Stalder and Ulmer 2001; Fabbrizio et al. 2013). All runs were terminated by switching off the power. After decompression, the charge was removed from the assembly, and the recovered capsule was weighed and pierced to check for potential leakage. When pierced, the pH of the extruding fluid was checked with indicator paper; the checked fluid was in all cases neutral (pH = 7), indicating that the possibly dissolved alkalis are balanced by chlorine, that Al acidic and basic species are negligible and that neutral complex such as NaAl(OH)_4_ may form. Subsequently, the pierced capsule was dried in an oven at 120 °C (upon drying, salt crystals formed at the opening of the capsule) and weighed again. Usually, the weight loss upon drying corresponds to 90–100 % of the initial water content. In the two samples buffered by the reactions Fe–FeO and Ni–NiO, remnant metal foil from the oxygen buffer material and brightly colored crystalline material was recovered from the capsule. The metal foil was analyzed by EDS during the EMPA session. All recovered capsules were embedded in epoxy and ground by hand. As soon as the trap became visible, the sample was impregnated with epoxy to prevent destruction during preparation. Later, the capsule was ground until its maximum cross section was exposed.

### Electron microprobe

**Fig. 1:**
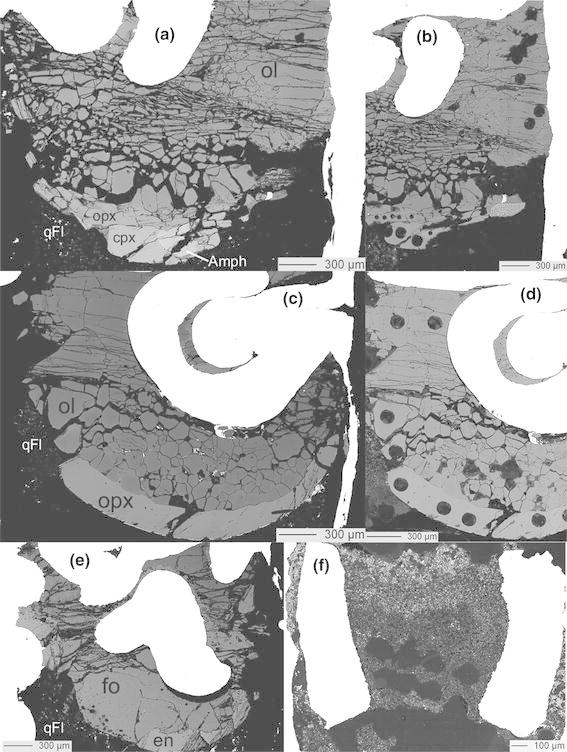
BSE images of **a** sample Cl-26, **b** sample Cl-26 with laser ablation spots in the crystalline phases, **c** sample Cl-24, **d** sample Cl-24 with laser ablation spots in the crystalline phases, **e** sample Cl–F-1, the one investigated by TEM, **f** diamond trap of sample Cl-22 after laser ablation analysis. The *circular holes* are laser ablation pits now filled by epoxy. *Ol* olivine (*dark gray*), *opx* orthopyroxene (*medium gray*), *cpx* clinopyroxene (*light gray*), *amph* amphibole (*lighter gray*), *en* enstatite (*light gray*), *fo* forsterite (*dark gray*), *qFl* quenched fluid

Electron microprobe measurements were taken using a Jeol Superprobe 8100 at Innsbruck University. The crystals were analyzed with an electron beam energy of 15 keV, a beam current of 10 nA, a focused beam and counting times of 20 s. Subsequently, Ti, Na, Cl and F were measured with a beam current of 400 nA and 240-s counting time on peak and 120 s on the background. A detailed explanation of the analytical protocol to measure chlorine concentrations, including a comparison with samples for which the Cl contents were independently determined, is reported in Fabbrizio et al. (2013). Quartz (Si), diopside (Ca), olivine (Mg), rutile (Ti), aegirine (Na), atacamite (Cl), corundum (Al), chromite (Cr), almandine (Fe), rhodonite (Mn), Ni-olivine (Ni) and F-topaz (F) were used as standards. Replicate analyses were carried out on the same sample (30–40 points in each sample) to check for compositional homogeneity. Limits of detection were 2 ppm for Cl, 6 ppm for Na, 9 ppm for Ti and 15 ppm for F. Associated relative errors (based on counting statistics) were less than 10 % for Cl, less than 5 % for Na, ~20 % for Ti and less than 1 % for F. Due to the overlap of the Fe*Lα* over the F*Kα*, fluorine was analyzed only in the Fe-free experiments. The BSE images show the absence of fluid inclusions inside the crystals and the absence of beam damage on the surface of the crystals after the electron microprobe analysis for Cl (Fig. 1).

### LA–ICP–MS

Element concentrations of the crystalline phases and of the fluid solute trapped between the diamonds were analyzed by LA–ICP–MS at the Laboratory for Inorganic Chemistry, ETH Zürich, using a 193-nm ArF excimer laser system (Günther et al. 1997) coupled to an ELAN 6100 quadrupole ICP–MS. All elements except Cl were standardized against a NIST SRM610 glass, and a synthetic Cl-bearing basaltic glass (Fabbrizio et al. 2013) was used as standard for Cl. The glass was analyzed two times before and two times after performing analyses of the samples of interest and served as external standard. Na was used as internal standard for the synthetic glass. Ba was used for internal standardization to calculate element concentration in the quenched fluid, by assuming that Ba (the most incompatible element in this study) partitions totally into the fluid phase.

Diamond traps (Fig. 1f) were measured with the laser imaged to 120 μm diameter at repetition rates of 5 Hz. Eight analyses were carried out on the diamond trap for each sample. In order to check the potential influence of mass interferences of ^23^Na^12^C and ^35^Cl, both chlorine isotopes (^35^Cl and ^37^Cl) were measured. Since the differences in the isotope ratio were always below 3 %, the influence of this interference was considered negligible. The detection limit for chlorine was below 100 ppm. For minerals analysis, the NIST SRM610 glass was used to calibrate elements. Element concentration calculations were based on the Si concentration as determined by electron microprobe analysis. For the analysis of the crystalline phases, the diameter of the laser was adjusted as function of the crystal size (Fig. 1b, d); in general, beam diameters of 50–120 μm were used at repetition rates of 5 Hz.

### IR spectroscopy

**Fig. 2:**
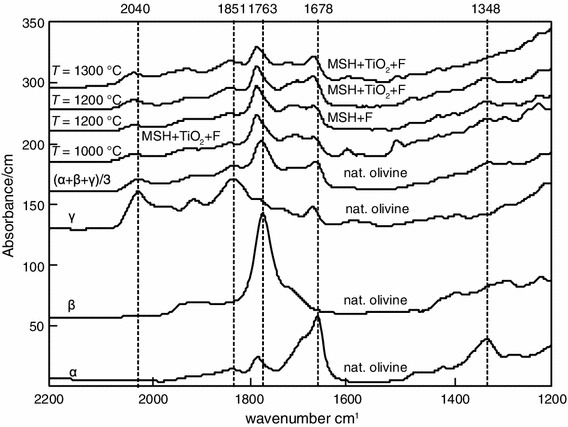
Polarized IR spectra of olivine sections parallel to α, β, γ and average spectrum (α + β+γ)/3. Unpolarized IR spectra of olivine averaged over 10–15 randomly oriented crystals from the MSH + F ± TiO_2_ system

All experimental charges were cut as whole capsule by a diamond wire saw to preserve the texture, and then, double-sided polished wafers of 150 μm thickness were prepared. Sample thicknesses were measured using a Mitutoyo micrometer and are accurate to ±2 μm. In order to evaluate the orientation of the measured olivine crystals, reference spectra on oriented olivine crystals from Stöppling/Germany (Oehm et al. 1983) were recorded that were later compared with the olivine spectra of the experimental charges (Fig. 2).

IR spectra were recorded at room temperature in transmission mode using a Bruker Vertex 70 FTIR spectrometer, which was continuously flushed with dried air to minimize water–vapor background, coupled to a Hyperion 3000 microscope equipped with (1) a usual nitrogen-cooled MCT-D316-025 (mercury cadmium telluride) detector (in the following called single-element detector) and (2) a nitrogen-cooled focal plane array (FPA) detector consisting of 64 × 64 MCT-D364 detectors. For measurements with the single-element detector, each spectrum was acquired by 64 scans in the 550–7,500 cm^−1^ range with a spectral resolution of 2 cm^−1^. The FPA detector enables FTIR imaging of an area measuring 170 × 170 μm with a lateral pixel resolution of 2.7 μm. For each spectrum, 64 scans in the 850–3,950 cm^−1^ range were acquired with a spectral resolution of 4 cm^−1^. Selected spectral ranges were integrated automatically for all spectra, and the results were color-coded and graphically displayed as a map that was used as overview for measurements with the IR single-element detector masked with apertures of typically 50 × 50 μm^2^. Grain boundaries and areas on the crystal, which are penetrated by cracks or inclusions, could be easily detected and avoided as analysis area for IR measurements. From each capsule and on each wafer, unpolarized spectra of 10–15 randomly oriented olivine single crystals were recorded and averaged.

### TEM

In order to check whether F in olivine is incorporated by F-clinohumite lamellae (Stalder and Ulmer 2001), transmission electron microscopy (TEM) was carried out on a forsterite of run Cl–F-1 using a FEI Tecnai G^2^ F^20^ X-Twin transmission electron microscope at GeoForschungsZentrum Potsdam, Germany, operated at 200 kV. Selected area electron diffraction (SAED), combined with bright field (BF), high-resolution (HR-TEM) and scanning transmission electron microscopy using a high-angle annular dark field detector in scanning transmission mode (STEM-HAADF), was performed to investigate the local defect structure of forsterite. The sample for TEM was prepared by first determining the orientation of the olivine using electron-backscattered diffraction (EBSD) with an Ultra 55 Plus (Carl Zeiss SMT) field emission scanning electron microscope. The foil for TEM was prepared by the focused ion beam technique (FIB) (Wirth 2004; Lee et al. 2003; Phaneuf 1999) using a FEI FIB200TEM focused ion beam device. After cutting the foil to the dimensions of 12 × 8 μm² and a thickness of about 100 nm, the foil was extracted from the excavation site using the lift-out technique (Giannuzzi et al. 1997). It was transferred to a copper TEM grid with a holey carbon foil to sustain the TEM foil using a manipulator.

## Results

### Textures of minerals and diamond traps

Experimental conditions and results are summarized in Tables 1, 2 and 3 and in the Online Resource 1. Since one of the aims of this study was the determination of mineral/fluid partition coefficients, it is important to judge whether crystals grew subsolidus (and consequently the term ‘fluid’ can be used) or supersolidus, i.e., in the presence of a hydrous melt. The simple system forsterite–enstatite–water approaches melting at 1,400 °C and 2 GPa (Kushiro and Yoder 1969); however, the presence of NaCl suppresses melting because it lowers the water activity (Aranovich and Newton 1996, 1997; Shmulovich and Graham 1996). A Cl/(Cl + H_2_O) molar ratio of 0.2 and 0.4 increases the temperature of the solidus in the system Mg_2_SiO_4_ + MgSiO_3_ + H_2_O + KCl at 5 GPa, respectively, of ~200 and ~350 °C (Chu et al. 2011). Consequently, since the experiments reported here have a Cl/(Cl + H_2_O) molar ratio of 0.3, they can be considered being subsolidus. The textures of the experimental charges can serve as additional constraint of the absence of a melt during the runs. Textures of representative run products are shown in Fig. 1. No particular textural differences are noted between experiments performed at different temperatures (Fig. 1a–e). Phases interpreted to have crystallized in equilibrium with each other in the absence of a melt are euhedral to subhedral and typically have the same grain size. The quenched phase is subhedral and typically displays acicular habit. The small amount of quench phase (Fig. 1a–e) is interpreted to represent the solute quenched from the subsolidus fluid phase. No abrupt increase in the amount of quenched phase with temperature was observed, which is interpreted to indicate the absence of a melt in all performed experiments.

**Table 2:** Analyses of the run products (model system and natural peridotite) by EMPA

Experiment (*P*, GPa/*T*, °C)	F-2		F-3				Cl-F-1		F-1		
	(2/1300)		(2/1200)				(2/1200)		(2/1000)		
	fo	en	fo	en	chu	fo	en	fo	en	fo+tlc	tr
SiO_2_ (wt%)	43.20 (18)	60.58 (33)	40.75 (58)	58.99 (86)	36.66 (40)	42.95 (51)	58.72 (51)	42.44 (22)	59.84 (30)	49.63	59.10 (48)
TiO_2_	0.0015 (5)	0.03 (0)	0.03 (0.01)	0.01 (1)	0.97 (10)	–	–	–	0.06 (2)	0.40	0.24 (5)
Al_2_O_3_	–	–	–	–	–	–	–	–	–	–	–
Cr_2_O_3_	–	–	–	–	–	–	–	–	–	–	–
FeO	–	–	–	–	–	–	–	–	–	–	–
MnO	–	–	–	–	–	–	–	–	–	–	–
MgO	55.34 (21)	38.47 (25)	59.20 (38)	41.31 (19)	57.27 (40)	55.71 (23)	38.56 (30)	57.96 (31)	40.47 (14)	47.52	27.36 (95)
NiO	–	–	–	–	–	–	–	–	–	–	–
CaO	0.03 (0)	0.18 (2)	0.02 (1)	0.29 (13)	0.00	–	–	0.02 (1)	0.23 (10)	0.26	10.79 (7)
Na_2_O	–	–	–	–	–	0.03 (4)	0.06 (7)	–	–	–	–
Cl	–	–	–	–	–	0.017 (7)	0.003 (2)	–	–	–	–
F	0.00	0.00	0.077 (30)	0.00	3.06 (0.1)	0.090 (4)	0.00	0.00	0.00	3.14	2.04 (10)
Total	98.6 (3)	99.3 (4)	100.0 (9)	100.6 (9)	98.0 (5)	98.8 (5)	97.3 (5)	100.4 (3)	100.6 (4)	100.9	99.5 (5)
*D*_Cl_^mineral/startmix^						0.0016	0.00029				
*D*_F_^mineral/startmix^			0.16		6.25	0.096				6.41	4.16

Experiment (*P*, GPa/*T*, °C)	F-1	^a^F-5		^b^F-4	F-6		F-7	
	(2/1000)	(2/1300)		(2/1300)	(2/1300)		(2/1100)	
	di	ol	ox	ol	ol	opx	ol	opx
SiO_2_ (wt%)	55.31 (14)	38.26 (44)	0.38 (3)	39.98 (23)	42.03 (13)	58.94 (31)	41.90 (13)	57.90 (24)
TiO_2_	0.21 (4)	0.023 (3)	0.99 (9)	0.0033 (11)	0.0037 (9)	0.07 (2)	0.0053 (15)	0.10 (1)
Al_2_O_3_	–	0.09 (2)	1.24 (3)	0.01 (1)	0.00	0.38 (14)	0.00	0.52 (7)
Cr_2_O_3_	–	0.07 (4)	1.99 (34)	0.13 (7)	0.09 (4)	0.34 (7)	0.04 (3)	0.46 (23)
FeO	–	22.64 (93)	80.82 (57)	1.76 (38)	0.29 (5)	0.16 (6)	3.63 (24)	4.31 (7)
MnO	–	0.07 (2)	0.06 (3)	0.11 (2)	0.07 (2)	0.06 (2)	0.07 (2)	0.11 (2)
MgO	21.73 (37)	39.22 (79)	14.71 (16)	46.21 (22)	55.90 (32	38.23 (5)	53.31 (19)	34.32 (25)
NiO	–	0.02 (2)	0.12 (6)	12.07 (75)	0.06 (5)	0.03 (2)	0.32 (1)	0.13 (2)
CaO	22.66 (48)	0.34 (3)	0.02 (1)	0.09 (1)	0.05 (2)	0.44 (2)	0.06 (2)	1.27 (12)
Na_2_O	–	0.02 (2)	0.10 (6)	0.04 (2)	0.05 (3)	0.13 (1)	0.04 (2)	0.20 (3)
Cl	–	0.0014 (5)	na	0.0015 (5)	0.0015 (5)	0.0019 (7)	0.0056 (20)	0.0034 (7)
F	0.069 (16)	na	na	na	na	na	na	na
Total	100.0	100.7 (5)	100.4 (1)	100.4 (4)	98.6 (3)	98.8 (4)	99.39 (26)	99.33 (28)
*D*_Cl_^mineral/startmix^		0.00013		0.00014		0.00018	0.00053	0.00032
*D*_F_^mineral/startmix^	0.14							

^a^ Capsule lined with Fe foil

^b^ Capsule lined with Ni foil

**Table 3:** Mineral/fluid partition coefficients

Sample (*P*, GPa/*T*, °C)	2/13,00	2/1,200	2/1,100		2/1,000			2/900			
Mineral	ol	ol	ol	opx	ol	opx	cpx	ol	opx	cpx	amph
Cl	9 × 10^−5^ (2)	1.8 × 10^−5^ (16)	2 × 10^−4^ (0)	1.7 × 10^−4^ (8)	1.8 × 10^−4^ (8)	3.7 × 10^−4^ (3)	2 × 10^−4^ (1)	1.5 × 10^−4^ (3)	1.5 × 10^−4^ (4)	1.4 × 10^−4^ (2)	0.0049 (3)
Li	0.13 (4)	0.07 (3)	0.33 (13)	0.31 (9)	0.27 (10)	0.18 (6)	0.25 (11)	0.35 (7)	0.48 (7)	0.72 (19)	0.82 (30)
Na	0.0048 (2)	0.0018 (2)	0.0020 (1)	0.018 (2)	0.0037 (24)	0.0020 (0)	0.27 (2)	0.0028 (4)	0.018 (3)	0.28 (1)	0.63 (0)
Rb	0.012 (11)	–	–	0.008 (5)	0.0018 (8)	–	–	–	–	–	–
Sr	0.007 (4)	0.00011 (8)	–	0.0018 (9)	0.006 (4)	0.0021 (14)	0.23 (12)	0.0028 (19)	0.0034 (23)	0.21 (3)	0.26 (7)
Ca	0.012 (3)	0.005 (2)	0.016 (8)	0.58 (24)	0.049 (39)	0.96 (73)	19 (13)	0.025 (11)	0.78 (34)	20 (8)	7 (3)
Mg	3.30 (38)	9 (3)	38 (9)	24 (6)	81 (37)	51 (23)	24 (11)	122 (93)	77 (59)	34 (26)	39 (30)
Mn	0.46 (8)	0.78 (28)	1.17 (57)	4 (2)	2 (1)	4 (2)	4 (2)	1.4 (1)	4.1 (1)	5.2 (5)	3.82 (22)
Fe	13 (4)	7 (4)	15 (7)	32 (14)	6 (1)	10 (2)	13 (2)	32 (9)	60 (15)	92 (23)	109 (26)
Ni	113 (32)	45 (27)	113 (71)	50 (32)	243 (113)	94 (44)	52 (24)	175 (15)	80 (7)	58 (5)	120 (23)
B	0.022 (6)	0.085 (59)	0.83 (46)	0.8 (4)	0.073 (16)	–	–	0.50 (10)	–	–	0.58 (10)
Al	0.0045 (24)	0.004 (1)	0.015 (7)	0.86 (25)	0.10 (6)	4 (2)	14 (8)	0.053 (26)	3 (2)	10 (5)	35 (17)
Cr	0.75 (8)	1.75 (81)	12 (4)	4 (1)	5 (4)	24 (15)	6.5 (5)	21 (6)	15 (5)	2.71 (80)	2 (1)
Sc	0.13 (2)	0.28 (11)	1.38 (54)	23 (6)	9 (5)	76 (38)	427 (283)	–	–	–	–
La	–	–	–	–	–	–	0.77 (34)	–	–	0.56 (35)	0.93 (66)
Ce	0.0078 (40)	–	0.022 (15)	0.015 (9)	0.012 (9)	–	0.96 (62)	0.016 (11)	–	1.06 (38)	1.76 (77)
Pr	–	–	–	–	–	–	2 (1)	–	–	1.82 (84)	2.11 (93)
Nd	–	–	–	–	–	–	3 (2)	–	–	4 (2)	4 (2)
Lu	0.017 (4)	0.017 (6)	0.043 (23)	1.3 (6)	0.25 (17)	4 (3)	41 (29)	0.20 (8)	8 (3)	80 (30)	37 (14)
Y	–	–	–	0.41 (19)	–	0.27 (22)	6 (3)	–	0.54 (26)	16 (5)	6 (2)
Ti	0.009 (3)	0.009 (3)	0.022 (6)	0.51 (12)	0.17 (8)	4 (2)	14 (6)	0.14 (5)	4 (1)	4 (1)	12 (4)
Zr	–	–	–	0.042 (26)	–	–	–	–	–	15 (10)	36 (19)
V	0.0019 (4)	0.0018 (7)	0.0083 (36)	0.039 (17)	0.014 (14)	0.073 (65)	–	0.046 (27)	0.22 (12)	0.86 (14)	7 (1)
P	0.12 (2)	0.09 (3)	0.23 (10)	0.22 (10)	0.23 (5)	0.28 (7)	0.27 (7)	0.21 (9)	0.35 (17)	0.33 (14)	0.29 (12)
Nb	0.009 (3)	–	–	–	–	–	–	–	–	–	9 (4)
Ta	0.0086 (23)	–	0.08 (4)	–	–	–	–	–	–	–	–

### Phase assemblages

Run products were mainly composed of olivine, pyroxenes, salt and aqueous fluid. In single cases, additional phases such as clinohumite, amphibole or spinel were observed. Individual phase assemblages are listed in Table 1. The size of the crystals varied between 80 and 300 μm in the natural system and was over 300 μm in the MSH system (Fig. 1). Electron microprobe analyses are listed in the Online Resource 1 and in Table 2. No chemical zoning patterns were observed. In the experiments with the natural composition, only olivine (Fo_97_) is present at temperature above 1,100 °C, at 1,100 °C orthopyroxene appears, at 1,000 °C olivine coexists with ortho- and clinopyroxene and at 900 °C amphibole occurred in addition. Spinel coexists with olivine in the run buffered by the reaction Fe–FeO. The Cl concentrations in olivine are 20 ± 14 ppm, in opx 26 ± 10 ppm and in cpx 20 ± 4 ppm, whereas the Cl contents of amphibole is 585 ± 36 ppm.

**Fig. 3:**
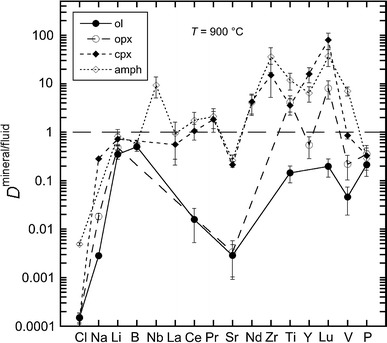
Representative mineral/fluid partition coefficients for coexisting minerals from experiments Cl-26. The *horizontal dashed line* represent the limit between compatibility (*D* ^mineral/fluid^ > 1) and incompatibility (*D* ^mineral/fluid^ < 1)

In the synthetic system, forsterite (Fo_100_) coexists with enstatite in all runs. At 1,200 °C, clinohumite coexists with forsterite and enstatite and at 1,000 °C F-tremolite (4.2 wt% F), diopside (Di_85_En_15_ containing 690 ppm F) and an unidentified F-bearing phase—supposedly talc—occurred. At 1,200 °C, forsterite has a F content of 900 ± 40 ppm and a Cl content of 170 ± 70 ppm. Enstatite shows a Cl content of 30 ± 20 ppm, similar to the opx in the run with natural starting composition. The aqueous fluids are homogeneous with respect to their major element composition. Most trace elements were below detection limit both in minerals and in the quenched solute. Concentrations measured with LA–ICP–MS and calculated partition coefficients (mineral/fluid) are illustrated in Fig. 3 and listed in the Online Resource 1 and in Table 3.

### Fluid compositions

The compositions of the aqueous fluids were determined with LA–ICP–MS (Online Resource 1). The aqueous fluids of each run are homogenous with respect to their major element composition and contain large amount of silicate components (mainly Si, Mg, Al, Ca, Na and Cl). Measured fluid compositions are generally consistent from run to run with the temperature of the run.

**Fig. 4:**
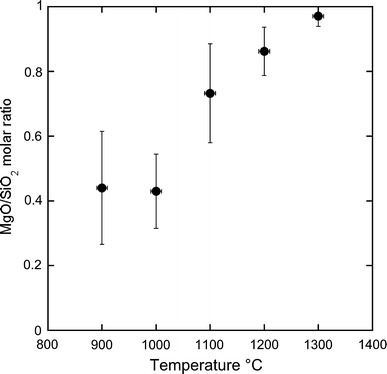
MgO/SiO_2_ molar ratio of the fluids calculated from the LA–ICP–MS analyses (Online Resource 1) versus the temperature (°C) of the runs

The plot of the MgO/SiO_2_ molar ratios of the fluids versus the temperature of the runs (Fig. 4) shows a positive correlation, probably reflecting a decreased solubility of silica with temperature.

### Mineral/fluid partition coefficients

Mineral/fluid partition coefficients (Fig. 3) show that Cl and the large ionic lithophile elements (LILE) always partition into the fluid phase, whereas most other trace elements behave compatible in clinopyroxene and amphibole, moderately incompatible to compatible in orthopyroxene and incompatible in olivine (Fig. 3). These findings, specifically the order *D* ^amph/fluid^ ≈ *D* ^cpx/fluid^ > *D* ^opx/fluid^ > *D* ^ol/fluid^, is in general agreement with previous studies (Mysen 1979; Brenan et al. 1995; Ayers et al. 1997; Stalder et al. 1998; Caciagli et al. 2011; Fabbrizio et al. 2013). In contrast, *D* ^mineral/fluid^ is very similar for Li and P for all observed silicate phases.

Measured olivine/fluid partition coefficients show that olivine strongly depletes Cl, Na, Ce and Sr relative to the fluid (Fig. 3), with low values for Cl ~10^−4^, intermediate values for Na and Sr (~10^−3^) and higher values for Ce (~10^−2^). Olivine/fluid values for Li, B, Ti, Lu, V and P are ~10^−1^.

Orthopyroxene/fluid values (Fig. 3) are given as follows: Cl ~10^−4^, Sr ~10^−3^, Na and Ce ~10^−2^, Li, Y, V and P ~10^−1^, Ti and Lu ~5.

Clinopyroxene/fluid partition coefficients show a strong enrichment of Pr, Nd, Zr, Ti, Y and Lu in clinopyroxene relative to the fluid (Fig. 3), with values for Lu ~10^2^, for Ti, Y, Nd, Pr and Zr in the range 1–10. Clinopyroxene/fluid values for Na, Li, La, Sr, V and P are in the range 10^−1^–1 and for Cl are ~10^−4^.

Amphibole is enriched in Nb, Ce, Pr, Nd, Zr, Ti, Y, Lu and V with respect to the fluid with amphibole/fluid values larger than 1 (Fig. 3). Amphibole/fluid values are in the range ~10^−1^–1 for Li, B, La, Sr, P and ~5 × 10^−3^ for Cl.

**Fig. 5:**
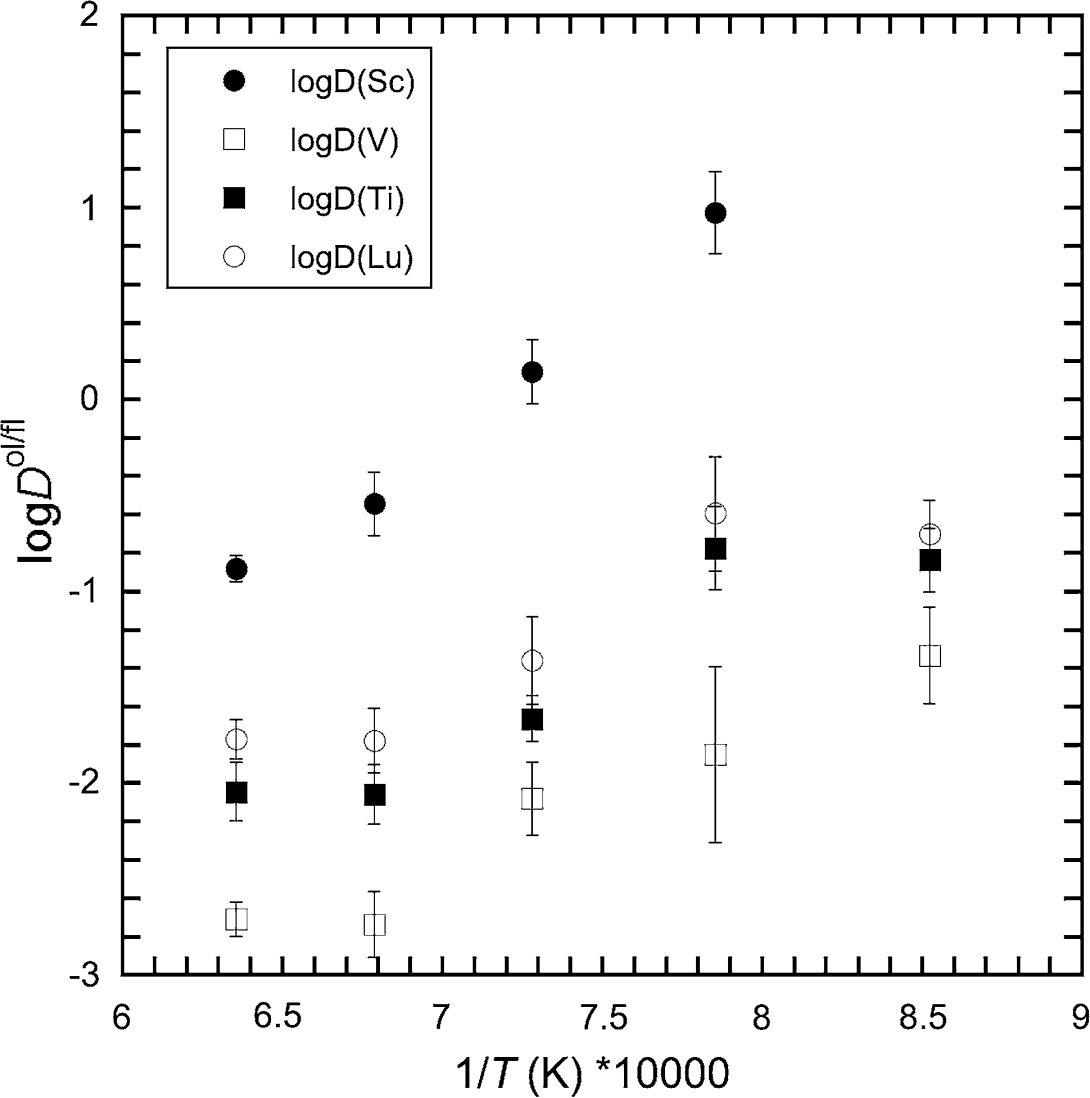
Partition coefficients log*D* ^ol/fluid^ for some trace elements as function of the inverse temperature (K)

One important factor controlling olivine-fluid trace element partitioning is the solute content of the fluid phase (Fig. 5). As observed in previous studies (Stalder et al. 2001; Kessel et al. 2004), the total solute content is positively correlated with temperature (Online Resource 1) concomitant with a decrease in *D* ^ol/fluid^. This trend is extremely pronounced for some cations (e.g., Ti), with *D* ^ol/fluid^ dropping by 1–2 log units as the solute content increases to a similar extent. Similar effects on *D* ^mineral/fluid^ were reported by Brenan and Watson (1991) and Brenan et al. (1995).

### Cl and F partition coefficients

Cl mineral/fluid partition coefficients (*D*_Cl_) were calculated from the Cl concentration in the mineral measured by EMPA and the Cl concentration of the coexisting fluid measured by LA–ICP–MS. The *D*_Cl_^ol/fl^ is between 9 × 10^−5^ and 2 × 10^−4^. Chlorine in orthopyroxene is more compatible than in olivine with *D*_Cl_^opx/fl^ values between 1.5 × 10^−4^ and 3.7 × 10^−4^. Clinopyroxene has *D*_Cl_^cpx/fl^ values of 1.4 × 10^−4^ and 2 × 10^−4^ similar to orthopyroxene, and amphibole shows the highest *D*_Cl_^amph/fl^ value of 4.9 × 10^−3^. For fluorine and for runs performed without diamond trap, the partition coefficients can be estimated as the ratio between the concentration in mineral versus the concentration in the starting mixture. We obtain *D*_F_^fo/startmix^ ~0.1 for forsterite characterized by the presence of humite-type point defects, *D*_F_^chu/startmix^ = 6.25 and *D*_F_^Di/startmix^ = 0.14 for diopsidic pyroxene (Table 2). The values for *D*_Cl_^ol/startmix^ are between 1.6 × 10^−3^ and 5.3 × 10^−4^ and that for *D*_Cl_^opx/startmix^ vary between 1.8 × 10^−4^ and 3.2 × 10^−4^ in close agreement with the calculated partition coefficients.

### IR spectra and IR images

**Fig. 6:**
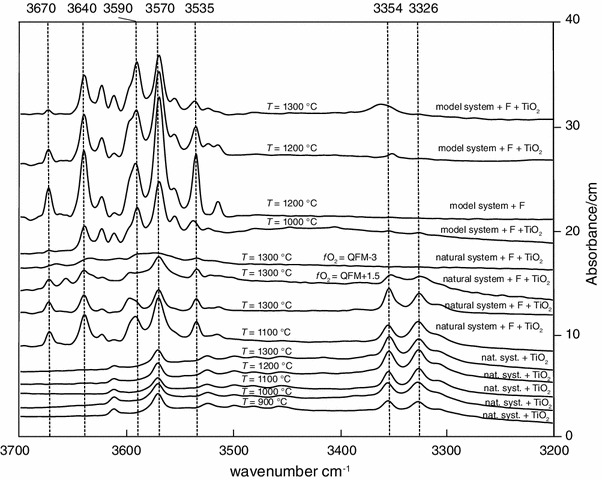
Unpolarized IR spectra of olivine averaged over 10–15 randomly oriented crystals for each run. Spectra are normalized to 1 cm thickness. *Vertical dashed lines* indicate the positions of OH bands. Experimental run details are labeled above each spectrum

**Fig. 7:**
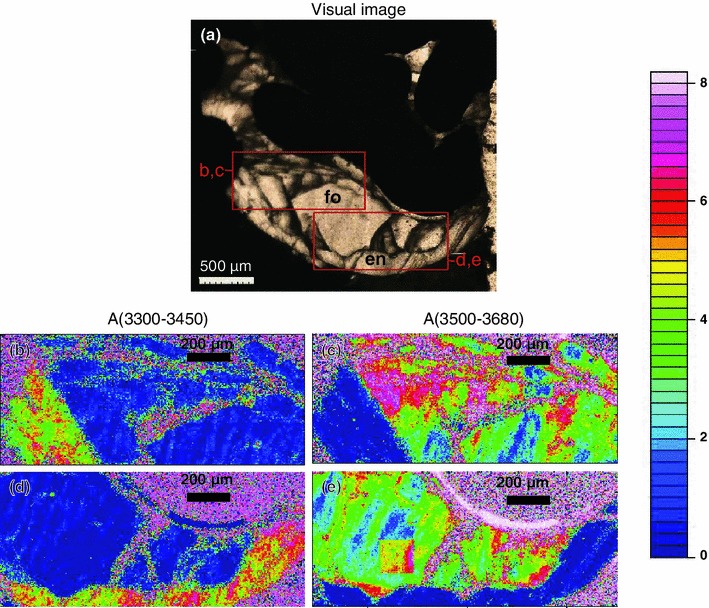
Visual image **a** of sample Cl–F-1, **b**–**e** IR images displaying total absorbances in the wavenumber range (see *color code* right) **b**, **d** 3,300–3,450 cm^−1^ and **c**, **e** 3,500–3,680 cm^−1^ *color-coded* according to the *scale bar* to the right (not normalized to thickness). Each pixel (2.7 × 2.7 μm^2^) contains the full information of an IR spectrum and can be extracted separately. Note the absence of any band close to 3,400 cm^−1^ indicating the absence of humite-type planar defects (Hermann et al. 2007). The *rectangular* feature *left* to the *center* in (**e**) represents one 170 × 170 μm^2^ frame. Compared to the surrounding frames, it exhibits enhanced absorbance interpreted as analytical artifact probably caused by an electric spike during data acquisition

Unpolarized IR spectra collected on olivine are shown in Fig. 6, and IR images that were recorded for visualization of the OH defects on the entire experimental charge are shown in Fig. 7.

Ti-bearing olivine crystals (Online Resource 1, Fig. 6), synthesized using the natural peridotite as starting material, exhibit absorption bands at 3,326, 3,354, 3,525, 3,571 and 3,612 cm^−1^. Olivine from the natural F-doped peridotite (Table 2; Fig. 6) displays also the absorption band at 3,535, 3,595, 3,640 and 3,670 cm^−1^, and the band at 3,570 cm^−1^ tends to become stronger (i.e., higher intensity). Olivine crystallized under relatively oxidizing conditions (*f*O_2_ = QFM + 1.5) shows an IR spectrum similar to that of olivine crystallized with no constrain on *f*O_2_, whereas the IR spectra of olivine crystallized under reducing conditions (*f*O_2_ = QFM − 3) do not have bands at 3,326 and 3,354 cm^−1^ and those over 3,500 cm^−1^ tend to be less intense. This observation supports the idea that the bands around 3,350 cm^−1^ can be assigned to Fe^3+^-related OH defects (Berry et al. 2007) and that olivine grown at low *f*O_2_ (below the Fe–FeO) contains much less water with respect to crystals from relatively more oxidized experiments (Grant et al. 2007b). Forsterite from the F-bearing model system (Table 2; Fig. 6) displays the strongest absorption bands at wavenumber similar to those for olivine from the natural peridotite plus F system. The main absorption bands are located at 3,569 and 3,536 cm^−1^, and the bands above 3,590 cm^−1^ are more intense with respect to the bands for olivine from the F-doped natural peridotite system. In addition, the bands at 3,326 and 3,354 cm^−1^ are absent. The thickness of the samples is comparable, and hence, the intensity of absorption is related to the amount of OH. The observed spectra indicate that the presence of F in the system permits to incorporate much more OH in olivine compared with olivine from the Ti-bearing and F-free system.

IR images (Fig. 7) were recorded on the whole sample Cl–F-1 containing the fluorine-rich forsterite crystals (Table 2; Fig. 6). No absorption bands close to 3,400 cm^−1^ were detected, suggesting the absence or a very low abundance of humite-type planar defects (Hermann et al. 2007).

Additional data on the amounts of water dissolved in olivine are reported in the Online Resource 2.

### TEM

**Fig. 8:**
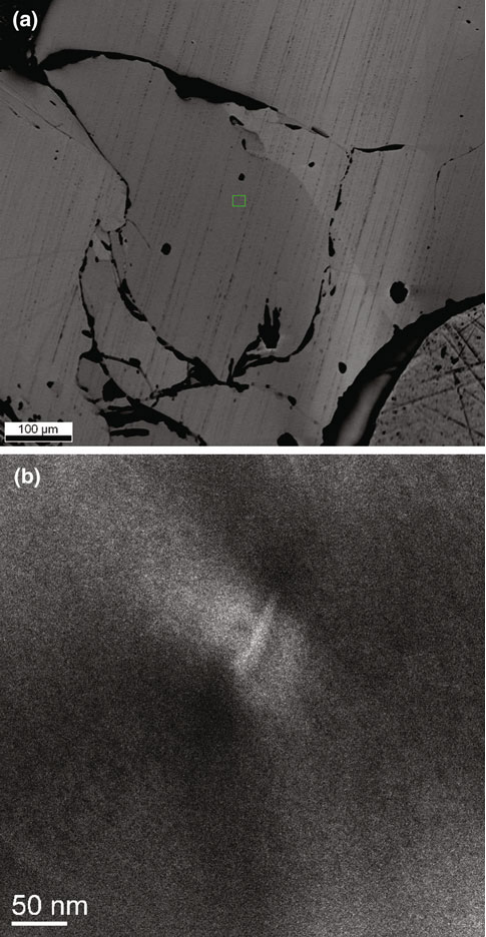
**a** Backscattered electron image of sample CL–F-1 showing lamellar features in the olivine crystals. The *rectangle* in the center depicts one of the regions from where EBSD measurements were taken. **b** *Bright* field image showing a dislocation in the olivine crystal

The analyzed TEM foils were cut from a forsterite crystal from sample Cl–F-1 with orientation ~[714] as determined by EBSD. This sample was chosen due to the high fluorine content of its forsterite (Table 2). The foils were cut after chemical polishing of the sample. Chemical polishing results in preferential material removal in regions with defects. Lamellar features that are approximately oriented in the direction expected for humite-type defects (parallel (001)_Ol_) are observed in the BSE image (Fig. 8a). Three TEM foils were cut, two across these features and one parallel, in order to determine its origin. Neither bright field TEM, nor HRTEM or STEM did show any chemical or structural variations in the investigated foils. As far as planar defects such as humite-lamellae are concerned, selected area diffraction in [100], [110] or [010] should depict evidence in the form of streaks, as humite has significantly different lattice parameters to olivine, always assuming the presence of enough such lamellae. The streaks in the diffraction pattern would be perpendicular to the plane of their orientation. Thus, the effect should best be visible on (001) diffraction spots, examples are given in Hermann et al. (2007), Kitamura et al. (1987), Risold et al. (2001), and Wirth et al. (2001). No such streaks were observed. Instead of lamellae, dislocations were observed in the bright field image (Fig. 8b), indicating another type of defect hitherto not considered as potential repository for halogens and hydrogen. However, the absence of planar defects cannot be proved, but the concentration is certainly rather low in this sample. Considering the fluorine content of the analyzed forsterite (0.09 wt% F) and the ideal fluorine content in stoichiometric F-clinohumite (6.1 wt% F), the hypothetical fraction of F-clinohumite hosted in the forsterite lattice is approximately 1.5%, corresponding to 0.34 mol% clinohumite in forsterite. For TEM observations, this would mean that only 1 out of 300 d-spacing perpendicular to the lamellae would appear broader.

## Discussion

### Halogen partitioning and major hosts for halogens in the mantle

Our single data point from experiment crystallizing forsterite with clinohumite defects gives *D*_ol_^F/Cl^ = 5.3, in agreement with the general observation that F is less incompatible than Cl in olivine (e.g., *D*_ol_^F/Cl^ values between 0.9 and 13 Hauri et al. 2006; Beyer et al. 2012; Dalou et al. 2012). Our results for chlorine partitioning between mantle minerals and coexisting aqueous fluid (Table 3) are in good agreement with results from the system forsterite–enstatite–pyrope–H_2_O–MgCl_2_ at 1,100 °C and 2.6 GPa (Bernini et al. 2013) and with those from the system MgO–SiO_2_–H_2_O–NaCl ± TiO_2_ ± Al_2_O_3_ at 900–1,300 °C and 2 GPa (Fabbrizio et al. 2013) having partition coefficients for *D*_Cl_^fo/fl^ and for *D*_Cl_^en/fl^ between 10^−5^ and 10^−3^.

The observed Cl concentrations in olivine crystals from this study are similar to the Cl content of olivine from high-pressure serpentinite and from metamorphic harzburgite, where Cl concentrations of 24–120 and 7–18 ppm, respectively, were detected (Scambelluri et al. 2004). Chlorine concentrations in natural olivine from spinel peridotites and oceanic basalts are lower and fairly homogeneous at 5.7 ± 0.5 ppm (Beyer et al. 2012). An implication of the present work is that halogens in the mantle may be incorporated in olivine as well as in other nominally anhydrous minerals (for example, pyroxenes) in addition to hydrous minerals such as amphibole, serpentine or chlorite. Taking into account the modal proportions in the mantle and estimations for the total halogen content (Saal et al. 2002), olivine may be regarded as an important host for halogens in the Earth’s mantle.

### Incorporation mechanism of halogens into nominally halogen-free minerals

In analogy to OH incorporation in olivine, two different groups of defects can be encountered for halogen incorporation in olivine: point defects (Berry et al. 2005, 2007) and planar defects (Kitamura et al. 1987; Hermann et al. 2007). Both groups of defects permit to incorporate OH in the forsterite structure and thus can serve as host for halogens by permitting a direct exchange between halogens and OH. Planar defects are more likely close to the clinohumite breakdown curve that depends on the *P*–*T*–*X* conditions (Weiss 1997; Ulmer and Trommsdorff 1999), but at conditions far above the clinohumite breakdown, OH and halogen incorporation is dominated by point defects (Hermann et al. 2007). Olivine IR absorption bands have been related to the type of defect permitting thus to distinguish between point and planar defects. IR absorption bands at 3,525 and 3,572 cm^−1^ have been assigned to humite-type point defects (Berry et al. 2005), whereas band at ~3,400 cm^−1^ is related to humite-type planar defects (Hermann et al. 2007). IR spectra recorded on F ± Ti-bearing olivine (Fig. 6) reveal strong absorption bands at 3,535 and ~3,570 cm^−1^, and no absorption band close to 3,400 cm^−1^ is revealed in the IR spectra of the same crystals and in the IR images (Fig. 7) of the fluorine-rich forsterite investigated by TEM, suggesting the presence of humite-type point defects instead of humite-type planar defects. Note that the bands at 3,535 and ~3,570 cm^−1^ tend to be less intense for F-free Ti-bearing olivine and for olivine coexisting with F-bearing minerals, whereas they tend to become more intense in the F-bearing systems at lower temperature. These observations suggest that in the F–Ti-bearing system, lower temperatures tend to promote the stabilization of point defects in olivine. In the olivine crystal investigated by TEM, no indications for humite-lamellae were found. This observation is in accord with the IR spectrum and images recorded on the same crystal, where no absorption bands around 3,400 cm^−1^ were detected, and—considering the relatively high synthesis temperature of 1,200 °C—the predominance of point defects was actually expected (Hermann et al. 2007). However, the observation of a dislocation may suggest that the concept of different defect regimes in the *P*–*T*–*X*-space is more complex than previously elaborated. It may be speculated that between the regimes of planar defects (at temperatures just above the clinohumite breakdown, Hermann et al. 2007) and point defects (far above clinohumite breakdown), a defect regime where dislocations prevail may exist.

## Electronic supplementary material

Below is the link to the electronic supplementary material.

Supplementary material Analyses of the run products (natural peridotite), major elements in minerals by EMPA, fluid and trace elements in minerals by LA-ICP-MS (DOC 61 kb)

Supplementary material Quantification of water dissolved in olivine (DOC 40 kb)
