# Supplementary material for: Experimental partitioning of halogens and other trace elements between olivine, pyroxenes, amphibole and aqueous fluid at 2 GPa and 900–1,300 °C
Source: Contrib Mineral Petrol. 2013 Jun 19;166(2):639–53. doi: 10.1007/s00410-013-0902-5 (PMC4459431; doi:10.1007/s00410-013-0902-5)
Supplement: Supplementary file 1 — Supplementary material Analyses of the run products (natural peridotite), major elements in minerals by EMPA, fluid and trace elements in minerals by LA-ICP-MS (DOC 61 kb) [file 410_2013_902_MOESM1_ESM.doc]

Online Resource 1. analyses of the run products (natural peridotite), major elements in minerals by EMPA, fluid and trace elements in minerals by LA-ICP-MS

**Title:** Experimental partitioning of halogens and other trace elements between olivine, pyroxenes, amphibole and aqueous fluid at 2 GPa and 900 to 1300 °C

**Journal name:** Contributions to Mineralogy and Petrology

**Authors:** Alessandro Fabbrizio, Roland Stalder, Kathrin Hametner, Detlef Günther, Katharina Marquardt

**Corresponding Author:** [Alessandro.Fabbrizio@uibk.ac.at](mailto:Alessandro.Fabbrizio@uibk.ac.at)

Institute of Mineralogy and Petrography, University of Innsbruck, Innrain 52f, 6020 Innsbruck, Austria

Experiment

(*P*, GPa/*T*, **°**C) (2/1300) (2/1200) (2/1100) (2/1000)

ol fl ol fl ol opx fl ol

SiO_2_ (wt%) 43.05 (26) 25.25 (90) 41.98 (82) 10.36 (90) 42.92 (40) 56.98 (28) 2.89 (97) 42.92 (28)

TiO_2_ na 0.70 (9) 0.00 0.54 (19) 0.01 (1) 0.12 (4) 0.23 (5) 0.00

Al_2_O_3_ na 3.05 (35) na 2.21 (80) bdl 0.76 (13) 0.81 (19) 0.01 (1)

Cr_2_O_3_ 0.09 (2) 0.11 (1) 0.09 (2) 0.04 (2) 0.09 (3) 0.06 (5) 0.01 (1) 0.09 (2)

FeO 2.96 (16) 0.23 (7) 2.81 (30) 0.43 (27) 2.73 (39) 5.72 (45) 0.18 (8) 3.46 (35)

MnO 0.07 (2) 0.14 (2) 0.06 (2) 0.07 (3) 0.06 (2) 0.18 (3) 0.05 (2) 0.07 (2)

MgO 54.24 (21) 16.44 (90) 53.90 (77) 5.99 (90) 53.91 (38) 33.84 (32) 1.42 (35) 53.26 (24)

NiO 0.32 (3) 0.00 0.32 (5) 0.01 (1) 0.28 (5) 0.15 (3) 0.00 0.31 (4)

CaO 0.03 (1) 2.24 (28) 0.04 (2) 3.23 (85) 0.03 (1) 0.75 (7) 1.29 (50) 0.03 (1)

Na_2_O 0.04 (1) - 0.04 (1) - 0.04 (1) 0.18 (2) - 0.03 (1)

Na^a^ 5.93 (37) 5.93 (56) 6.47 (65)

Cl 7.70 (89) 9.22 (95) 10.15 (30)

Na=Cl^b^ 5 5.99 6.59

NaCl^c^ 12.70 15.21 16.74

Na_2_O^d^  1.25 - -

F na 0.02 (1)

dissolved silicates^e^ 49.4 22.9 6.9

H_2_O^f^ 37.9 61.9 76.36

Total 100.8 (30) 100.0 99.2 (54) 100.0 100.1 (52) 98.7 (30) 100.0 100.2 (57)

Mg# 97 97 97 96

Cl (ppm) 7 (2) 17 (15) 20 (5) 18 (8) 19 (7)

Li 0.7 (0.1) 5 (1) 1 (0.1) 9 (3) 0.7 (0.2) 0.6 (0.1) 2 (0.6) 1 (0.1)

Na 302 (144) 593×10^2^ (3700) 107 (13) 593×10^2^ (5600) 132 (5) 1186 (108) 647×10^2^ (6500) 261 (166)

Rb 0.07 (0.06) 6 (3) bdl 18 (6) bdl 0.02 (0.01) 2 (1) 0.03 (0.01)

Sr 0.9 (0.6) 131 (18) 0.03 (0.02) 303 (131) 0.05 (0.04) 0.1 (0.03) 55 (22) 0.7 (0.2)

Ca 189 (43) 16×10^3^ (2000) 118 (4) 23×10^3^ (6100) 148 (48) 4229 (589) 9196 (3668) 329 (123)

Mg 327×10^3^ (6200) 99×10^3^ (5400) 312×10^3^ (2000) 36×10^3^ (6200) 327×10^3^ (5000) 206×10^3^ (1500) 8558 (2105) 322×10^3^ (10^4^)

Mn 490 (30) 1068 (180) 438 (30) 564 (202) 438 (112) 1470 (84) 374 (155) 699 (79)

Fe 226×10^2^ (1000) 1798 (578) 197×10^2^ (1100) 3325 (2077) 212×10^2^ (4000) 488×10^2^ (2300) 1404 (608) 313×10^2^ (4100)

Ni 2388 (69) 21 (6) 2114 (107) 47 (29) 2230 (58) 992 (73) 20 (12) 2508 (135)

Online Resource 1. continued

Experiment

(*P*, GPa/*T*, **°**C) (2/1300) (2/1200) (2/1100) (2/1000)

ol fl ol fl ol opx fl ol

B (ppm) 2 (0.3) 94 (22) 2 (0.6) 24 (15) 2 (0.6) 2 (0.4) 3 (1) 3 (0.3)

Al 72 (38) 16×10^3^ (1800) 43 (2) 117×10^2^ (4200) 63 (26) 3717 (659) 4310 (1000) 89 (23)

Cr 550 (11) 728 (80) 543 (56) 309 (139) 590 (74) 186 (30) 49 (14) 671 (214)

Sc 3 (0.3) 20 (2) 2 (0.2) 8 (3) 2 (0.7) 40 (3) 2 (0.5) 3 (0.3)

La bdl 1 (0.02) bdl 1 (0.5) bdl bdl 1 (0.4) bdl

Ce 0.02 (0.01) 3 (0.3) 0.05 (0.03) 2 (1) 0.03 (0.02) 0.02 (0.01) 1 (0.6) 0.03 (0.01)

Pr bdl 0.2 (0.05) bdl 0.3 (0.1) bdl bdl 0.2 (0.1) bdl

Nd bdl 1 (0.3) bdl 1 (0.5) bdl bdl 0.6 (0.2) bdl

Sm bdl bdl bdl bdl bdl bdl bdl bdl

Eu bdl bdl bdl bdl bdl bdl bdl bdl

Gd bdl bdl bdl bdl bdl bdl bdl bdl

Tb bdl bdl bdl bdl bdl bdl bdl bdl

Dy bdl bdl bdl bdl bdl bdl bdl 0.04 (0.01)

Ho bdl bdl bdl bdl bdl bdl bdl bdl

Er bdl bdl bdl bdl bdl 0.05 (0.01) bdl bdl

Tm bdl bdl bdl bdl bdl 0.01 (0.01) bdl bdl

Yb bdl bdl bdl bdl bdl 0.1 (0.03) bdl bdl

Lu 1.6 (0.3) 95 (13) 1 (0.1) 65 (24) 1 (0.4) 40 (9) 30 (12) 2 (0.3)

Y bdl 1 (0.2) bdl 1 (0.5) bdl 0.2 (0.05) 0.5 (0.2) bdl

Ti 37 (12) 4174 (529) 28 (2) 3232 (1122) 30 (5) 708 (55) 1390 (305) 45 (10)

Zr bdl 6 (1) bdl 5 (2) bdl 0.1 (0.04) 2 (1) 0.05 (0.01)

Hf bdl bdl bdl bdl bdl bdl bdl bdl

V 0.1 (0.02) 50 (3) 0.07 (0.02) 40 (12) 0.1 (0.01) 0.5 (0.1) 12 (5) 0.3 (0.2)

P 24 (1) 207 (34) 20 (2) 226 (73) 26 (2) 25 (2) 110 (48) 26 (1)

Nb 0.03 (0.01) 4 (0.5) bdl 4 (1) bdl bdl 2 (0.4) bdl

Ta 0.02 (0.01) 3 (0.5) bdl 2 (1) 0.04 (0.02) bdl 0.5 (0.1) bdl

Online Resource 1. continued

Experiment

(*P*, GPa/*T*, **°**C) (2/1000) (2/900)

opx cpx fl ol opx cpx amph fl

SiO_2_ (wt%) 57.04 (47) 53.15 (28) 2.29 (64) 43.33 (25) 57.19 (43) 53.23 (81) 48.96 (59) 1.49 (27)

TiO_2_ 0.15 (3) 0.65 (8) 0.05 (2) 0.00 0.13 (6) 0.24 (19) 0.44 (12) 0.03 (1)

Al_2_O_3_ 0.73 (12) 2.06 (57) 0.16 (9) 0.00 0.71 (32) 2.09 (58) 7.52 (28) 0.22 (10)

Cr_2_O_3_ 0.49 (19) 0.13 (7) 0.02 (1) 0.07 (3) 0.07 (4) 0.02 (4) 0.01 (2) 0.00

FeO 5.21 (89) 7.30 (22) 0.54 (6) 2.82 (38) 5.26 (41) 8.10 (79) 9.62 (19) 0.09 (2)

MnO 0.16 (5) 0.17 (7) 0.04 (2) 0.07 (3) 0.17 (5) 0.21 (4) 0.17 (2) 0.04 (0.01)

MgO 33.72 (56) 15.75 (68) 0.66 (30) 53.64 (30) 34.11 (42) 14.83 (48) 17.22 (30) 0.44 (0.34)

NiO 0.12 (4) 0.11 (3) 0.00 0.31 (3) 0.13 (4) 0.11 (2) 0.21 (8) 0.00

CaO 0.91 (24) 17.85 (74) 0.95 (67) 0.02 (1) 0.70 (15) 16.86 (71) 6.06 (22) 0.86 (37)

Na_2_O 0.20 (2) 2.37 (20) - 0.03 (2) 0.18 (3) 2.92 (64) 6.12 (4) -

Na^a^ 6.93 (67) 7.16 (63)

Cl 10.74 (90) 0.00 0.00 0.00 0.06 (0.01) 11.91 (83)

Na=Cl^b^ 6.97 7.73

NaCl^c^ 17.71 19.64

Na_2_O^d^ - -

F 0.01 (0.004) 0.04 (0.01) 0.02 (0.01) 0.05 (1) 0.08 (0.01)

dissolved silicates^e^ 4.7 3.2

H_2_O^f^ 77.6 77.2

Total 98.7 (50) 99.5 (15) 100.0 100.3 98.6 (26) 98.6 (34) 96.3 (68) 100.0

Mg# 97

Cl (ppm) 40 (26) 22 (9) 18 (4) 18 (5) 17 (3) 585 (36)

Li 1 (0.1) 1 (0.3) 4 (1) 0.7 (0.1) 1 (0.1) 1 (0.3) 1 (0.5) 2 (0.3)

Na 1425 (21) 188×10^2^ (1200) 693×10^2^ (6700) 204 (26) 1865 (440) 202×10^2^ (600) 453×10^2^ (2700) 716×10^2^ (6300)

Rb bdl bdl 17 (7) bdl bdl bdl 0.2 (0.1) bdl

Sr 0.2 (0.1) 26 (2) 117 (64) 0.2 (0.1) 0.3 (0.2) 18 (1) 21 (5) 82 (10)

Ca 6576 (2527) 114×10^3^ (10^4^) 6791 (4789) 155 (7) 4755 (342) 108×10^3^ (2600) 355×10^2^ (3200) 6117 (2645)

Mg 215×10^3^ (7700) 113×10^3^ (1700) 3981 (1809) 329×10^3^ (4200) 230×10^3^ (4800) 130×10^3^ (5129) 999×10^2^ (6400) 2660 (2038)

Mn 1435 (332) 1347 (85) 317 (155) 435 (29) 1272 (29) 1617 (162) 1183 (57) 310 (9)

Fe 495×10^2^ (9600) 647×10^2^ (1200) 4196 (466) 199×10^2^ (900) 484×10^2^ (800) 646×10^2^ (1300) 835×10^2^ (6100) 685 (161)

Ni 969 (57) 539 (37) 10 (5) 2262 (44) 1033 (38) 743 (15) 1555 (276) 13 (1)

Online Resource 1. continued

Experiment

(*P*, GPa/*T*, **°**C) (2/1000) (2/900)

opx cpx fl ol opx cpx amph fl

B (ppm) bdl bdl 39 (7) 3 (0.5) bdl bdl 3 (0.5) 5 (0.2)

Al 3131 (512) 121×10^2^ (2500) 847 (476) 61 (5) 3871 (38) 8505 (1361) 40×10^3^ (1800) 1153 (554)

Cr 2735 (2185) 568 (373) 126 (95) 625 (61) 452 (71) 79 (10) 59 (24) 29 (8)

Sc 27 (3) 153 (69) 0.4 (0.2) 2 (0.2) 49 (4) 59 (3) 7 (2) bdl

La bdl 0.6 (0.2) 0.8 (0.3) bdl bdl 0.6 (0.1) 1 (0.4) 1 (0.6)

Ce bdl 2 (0.5) 2 (1) 0.03 (0.02) bdl 2 (0.4) 3 (1) 2 (0.7)

Pr bdl 0.3 (0.1) 0.2 (0.1) bdl bdl 0.4 (0.06) 0.4 (0.03) 0.2 (0.1)

Nd bdl 2 (0.4) 0.7 (0.3) bdl bdl 2 (0.3) 2 (0.3) 0.6 (0.3)

Sm bdl 0.5 (0.1) bdl bdl bdl bdl 0.5 (0.1) bdl

Eu bdl 0.3 (0.1) bdl bdl bdl 0.2 (0.1) 0.1 (0.01) bdl

Gd bdl 0.5 (0.1) bdl bdl bdl 0.8 (0.3) 0.4 (0.1) bdl

Tb bdl 0.1 (0.02) bdl bdl bdl 0.1 (0.05) 0.1 (0.01) bdl

Dy bdl 0.8 (0.3) bdl bdl bdl 1 (0.1) 0.4 (0.1) bdl

Ho bdl 0.1 (0.06) bdl bdl bdl 0.2 (0.05) 0.1 (0.02) bdl

Er bdl 0.6 (0.2) bdl bdl bdl 0.9 (0.2) 0.3 (0.01) bdl

Tm bdl 0.1 (0.01) bdl bdl bdl 0.1 (0.04) 0.04 (0.01) bdl

Yb bdl 0.9 (0.1) bdl bdl bdl 2 (0.4) 0.6 (0.03) bdl

Lu 29 (13) 331 (85) 8 (5) 1 (0.2) 52 (8) 517 (20) 237 (21) 6 (2)

Y 0.2 (0.1) 4 (1) 0.6 (0.3) bdl 0.2 (0.1) 6 (0.2) 2 (0.2) 0.4 (0.1)

Ti 967 (173) 3803 (202) 270 (121) 30 (2) 845 (15) 734 (7) 2430 (177) 206 (76)

Zr bdl 5 (1) 0.3 (0.2) bdl bdl 7 (3) 16 (4) 0.4 (0.2)

Hf bdl 0.3 (0.1) bdl bdl bdl bdl 0.5 (0.1) bdl

V 2 (0.6) 13 (4) 22 (18) 0.2 (0.1) 1 (0.6) 4 (1) 35 (7) 5 (0.3)

P 31 (3) 30 (3) 112 (26) 25 (2) 42 (11) 39 (1) 34 (3) 119 (50)

Nb bdl bdl 1 (0.8) bdl bdl bdl 4 (1) 0.5 (0.2)

Ta bdl 0.2 (0.1) bdl bdl bdl 0.3 (0.2) 5 (0.4) bdl

ol: olivine, opx: orthopyroxene, cpx: clinopyroxene, amph: amphibole, fl: fluid, na: not analyzed, bdl: below detection limit

^a^Na content of the fluid by LA-ICP-MS. ^b^Assuming that Na = Cl (mole). ^c^NaCl: sum of (b) and Cl content. ^d^Excess of Na dissolved in the fluid as Na_2_O. ^e^Dissolved silicates: sum of the oxides dissolved in the fluid. ^g^Water content: (100 – dissolved silicate – NaCl)
