# Supplementary material for: Experimental partitioning of halogens and other trace elements between olivine, pyroxenes, amphibole and aqueous fluid at 2 GPa and 900–1,300 °C
Source: Contrib Mineral Petrol. 2013 Jun 19;166(2):639–53. doi: 10.1007/s00410-013-0902-5 (PMC4459431; doi:10.1007/s00410-013-0902-5)
Supplement: Supplementary file 2 — Supplementary material Quantification of water dissolved in olivine (DOC 40 kb) [file 410_2013_902_MOESM2_ESM.doc]

**Title:** Experimental partitioning of halogens and other trace elements between olivine, pyroxenes, amphibole and aqueous fluid at 2 GPa and 900 to 1300 °C

**Journal name:** Contributions to Mineralogy and Petrology

**Authors:** Alessandro Fabbrizio, Roland Stalder, Kathrin Hametner, Detlef Günther, Katharina Marquardt

**Corresponding Author:** [Alessandro.Fabbrizio@uibk.ac.at](mailto:Alessandro.Fabbrizio@uibk.ac.at)

Institute of Mineralogy and Petrography, University of Innsbruck, Innrain 52f, 6020 Innsbruck, Austria

Online Resource 2 - Water contents in olivine crystals

Since 10-15 unpolarized spectra in each run were collected for the synthesized olivine the water contents were quantified applying the method of Kovacs et al. (2008). The amounts of water dissolved in olivine were calculated using the calibrations of Libowitzky and Rossman (1997) and Bell et al. (1995).

Water concentrations (ppm) for olivine

| Run | H_2_O^a^ | H_2_O^b^ |
| --- | --- | --- |
| Cl-22 | 49 | 34 |
| Cl-23 | 53 | 37 |
| Cl-24 | 43 | 29 |
| Cl-25 | 52 | 43 |
| Cl-26 | 43 | 38 |
| F-2 | 77 | 112 |
| F-3 | 171 | 207 |
| Cl-F-1 | 180 | 234 |
| F-1 | 100 | 122 |
| F-5 | 34 | 50 |
| F-4 | 72 | 87 |
| F-6 | 50 | 55 |
| F-7 | 160 | 160 |

^a^Water concentrations (ppm) of olivine calculated using the Bell et al. (1995) calibration. ^b^Water concentrations (ppm) of olivne calculated using the Libowitzky and Rossman (1997) calibration.

Water concentrations in olivine are related to the presence of fluorine in the bulk composition permitting thus the OH incorporation via the stabilization of humite-point defects (Fig. 5). For comparison in the simplest system MgO-SiO_2_-H_2_O±TiO_2_, in absence of fluorine, forsterite crystals with a Ti content in the range of 25-35 ppm contain between 18 and 35 wt ppm H_2_O, whereas Ti-free forsterite have less than 10 wt ppm H_2_O (Fabbrizio et al. 2013).

References not listed in the manuscript

Bell DR, Ihinger PD, Rossman GR (1995) Quantitative analysis of trace OH

in garnet and pyroxenes. Am Mineral 80:465-474

Libowitzky E Rossman GR (1997) An IR absorption calibration for water in

minerals. Am Mineral 82:1111-1115

Kovács I, Hermann J, O’Neill HStC, Gerald JF, Sambridge M, Horváth G

(2008) Quantitative absorbance spectroscopy with unpolarized light: Part II. Experimental evaluation and development of a protocol for quantitative analysis of mineral IR spectra. Am Mineral 93:765-778
